# Oleanolic Acid Improves Obesity-Related Inflammation and Insulin Resistance by Regulating Macrophages Activation

**DOI:** 10.3389/fphar.2021.697483

**Published:** 2021-07-30

**Authors:** Wanqing Li, Hongxiang Zeng, Min Xu, Chenglong Huang, Linfen Tao, Jun Li, Ting Zhang, Hong Chen, Jing Xia, Chunli Li, Xi Li

**Affiliations:** Institute of Life Sciences, Chongqing Medical University, Chongqing, China

**Keywords:** oleanolic acid, obesity, inflammation, macrophages, ROS

## Abstract

The chronic low-grade inflammation of adipose tissues, primarily mediated by adipose tissue macrophages (ATMs), is the key pathogenic link between obesity and metabolic disorders. Oleanolic acid (OA) is a natural triterpenoid possessing anti-diabetic and anti-inflammation effects, but the machinery is poorly understood. This study investigated the detailed mechanisms of OA on adipose tissue inflammation in obese mice. C57BL/6J mice were fed with high-fat diet (HFD) for 12 weeks, then daily intragastric administrated with vehicle, 25 and 50 mg/kg OA for 4 weeks. Comparing with vehicle, OA administration in obese mice greatly improved insulin resistance, and reduced adipose tissue hypertrophy, ATM infiltration as well as the M1/M2 ratio. The pro-inflammatory markers were significantly down-regulated by OA in both adipose tissue of obese mice and RAW264.7 macrophages treated with interferon gamma/lipopolysaccharide (IFN-γ/LPS). Furthermore, it was found that OA suppressed activation of mitogen-activated protein kinase (MAPK) signaling and NACHT, LRR, and PYD domain-containing protein 3 (NLRP3) inflammasome through decreasing voltage dependent anion channels (VDAC) expression and reactive oxygen species (ROS) production. This is the first report that oleanolic acid exerts its benefits by affecting mitochondrial function and macrophage activation.

## Introduction

In recent years, obesity has become a seriously global threat to human health, which also lowers the quality of people’s life. The association of obesity with chronic inflammation contributes to a variety of metabolic diseases such as atherosclerosis, cardiovascular disease, insulin resistance (IR) and type 2 diabetes (Xu et al., 2003; Fernández-Sánchez et al., 2011; Yao et al., 2017). It has been found that obesity-induced inflammation begins with white adipose tissue (WAT), accompanied with the steady development to IR, and eventually the inflammation become systemic (Xu et al., 2003). Immune cells, especially macrophages, are key players in this development of inflammation in obese individuals (Shoelson et al., 2007). In obese mice, the up-regulation of macrophage-related genes is mainly induced in WAT (Xu et al., 2003). The adipose tissue macrophage (ATM) is essential in the pathogenesis of obesity and related metabolic disorders, both in genetic and diet-induced overweight rodents and obese patients (Weisberg et al., 2003).

ATMs account for only 10% of the total cells of the normal adipose tissue, while as high as 41% for the obese tissue (Weisberg et al., 2003). In lean mice, the majority of ATMs consist of F4/80^+^CD11b^+^CD206^+^ macrophages, also defined as the alternatively activated M2 macrophages. Differently, the main population of ATMs in obese mice is F4/80^+^CD11b^+^CD11c^+^ macrophages, known as the classically activated M1 macrophages (Lumeng et al., 2008). M1 macrophages are the main source of inducible nitric oxide synthase (iNOS) (Dallaire et al., 2008) and pro-inflammatory cytokines such as interleukin-6 (IL-6), tumor necrosis factor-alpha (TNF-α) (Lumeng et al., 2007a), in adipose tissues (Hill et al., 2014). M2 macrophages secrete anti-inflammatory cytokines such as arginase 1 (ARG1), mannose receptor C type 1 like protein 1 (MRC1) and IL-10 (Xiao et al., 2016). A large number of M1 macrophages can form a crown-like structure (CLS) around dying adipocytes in obese individuals (Lumeng et al., 2008). This formation is associated to the obesity-related IR and other processes. Several lines of evidence indicate that the obesity-related IR and inflammation can be improved by reduction of ATM infiltration or alternation of ATM polarization (Zhao et al., 2015; Zhuge et al., 2016; Jing et al., 2017; Ni et al., 2020). The specific depletion of M1 macrophages restores insulin sensitivity in diet-induced obese mice (David et al., 2008), whereas reducing the number of M2 macrophages predisposes lean mice to IR (Odegaard et al., 2007). Therefore, reducing macrophage infiltration in epidydimal white adipose tissue (eWAT) or inhibiting M1 polarization of ATMs can be a potential direction for seeking novel therapeutic targets for IR.

Chronic inflammation and oxidative stress reinforce each other in obesity. For example, macrophage infiltration into WAT leads to up-regulation of lipolysis (Samuel and Shulman, 2016), resulting in an increase of free fatty acid (FFA) and consequently reactive oxygen species (ROS) overproduction (Hurrle and Hsu, 2017). This overproduction results in oxidative stress by overwhelming the cellular antioxidant defense system (Furukawa et al., 2004), and promotes pro-inflammatory M1 macrophage activation, followed by induction of TNFα and monocyte chemoattractant protein-1 (MCP1) (Cruz et al., 2007; Lumeng et al., 2007b). Furthermore, this impairs the insulin signaling (Manna and Jain, 2015; Tangvarasittichai, 2015) and activates cellular stress-sensitive pathways such as mitogen-activated protein kinases (MAPKs) (Gan et al., 2016; Zhao et al., 2019), NACHT, LRR, and PYD domain-containing protein 3 (NLRP3) inflammasome (Sorbara and Girardin, 2011), etc. Many factors can act to expression of ROS to regulate oxidative stress and inflammation, and voltage dependent anion channels (VDAC) is one of them (Zhou et al., 2011).

As a natural triterpenoid, oleanolic acid (OA) widely exists in a variety of fruits such as apples, grapes, dates and pomegranates, as well as olive oil (Fai and Tao, 2009). Recent studies have shown OA’s pleiotropic benefits. For example, OA has been applied as an over-the-counter drug against human liver disease in China (Pollier and Goossens, 2012). OA has also been proved to have hypolipidemic (Wang et al., 2013), antioxidant (Su et al., 2018) and anti-inflammatory (Lee et al., 2013) activities, with a reductive effect on IR. Several recent studies reported that OA treatment decreased serum levels and gene expression of the pro-inflammatory cytokines in mice with obesity or related metabolic diseases (Zohra et al., 2018; Gamede et al., 2019; Matumba et al., 2019), but little is known whether OA improves inflammation in adipose tissue, and the underlying mechanisms have yet to be elicited. Besides, all the studies on improving IR mentioned above are animal models of prophylactic administration.

In this study, we demonstrated that OA improved HFD-induced IR, oxidative stress, adipocyte hypertrophy and adipose chronic inflammation in therapeutic administration mice model. The anti-inflammatory effects of OA were associated with reduction of ATM infiltration and its polarization to M1. OA attenuated the expression of interferon gamma/lipopolysaccharide (IFN-γ/LPS)-induced M1 marker genes in RAW 264.7 macrophages, presumably by reducing VDAC expression and ROS production to inhibit activation of MAPK signaling and NLRP3 inflammasome. These data suggest that in diet-induced obese mice, OA attenuates oxidative stress and chronic inflammation in the adipose tissue, thereby preventing IR.

## Materials and Methods

### Animals

Animal experiments were performed in accordance with the National Institutes of Health Guide for the Care and Use of Laboratory Animals with approval from the Ethics Committee of Chongqing Medical University. C57BL/6J male mice (4–6 weeks old, *n* = 21) were maintained under standard recommended conditions in the Laboratory Animal Center of Chongqing Medical University. Briefly, mice were housed in colony cages under 12-h light-dark cycles at 23 ± 1°C. Animals were on the standard diet for 2 weeks prior to the experiments, and then the mice were fed with a high-fat diet (HFD) (60% fat; Research Diets, United States) for 12 weeks. After that, the body weight curve of these mice were compared to that of the control mice on normal diet (ND), and glucose tolerance test (GTT) was performed to evaluate the success of obesity model. Then the mice were randomly divided into three groups (*n* = 7 per group). The groups were further fed on high fat diet while receiving vehicle (5% Gum Arabic solution) (Sangon, China, dissolved in ddH_2_O), OA 25 mg/kg or OA 50 mg/kg (selleck, United States, suspended in 5% Gum Arabic solution) per day individually by intragastric administration for 4 weeks, followed by a measurement of mice’s body weight and GTT. Finally, the mice were overnight fasted, assessed for the level of fasting blood glucose (FBG), and then sacrificed. The blood and WAT samples were collected for the follow-up experiments.

### Glucose Tolerance Test

The mice were fasted for 14 h for GTT, followed by measurements of body weight and FBG. Mice were then injected intraperitoneally with glucose solution. The blood glucose levels at 30, 60, 90 and 120 min were monitored. Notably, the obese mice fed with HFD for 12 weeks were administered with 50% glucose solution (2 mg/g body weight) while the obese mice intragastric administrated for 4 weeks received 25% glucose solution (1 mg/g body weight), due to the fact that the mice at this stage were more obese and their blood glucose levels were prone to exceed the detection limit).

### Cell Culture

RAW264.7 (ATCC, United States) macrophages were grown in DMEM (Gibco, United States) supplemented with 10% fetal bovine serum (FBS) (Gibco), 1% penicillin/streptomycin (P/S) (Beyotime Biotechnology, China) at 37°C with 5% CO_2_.

Femurs and tibias were isolated from male C57BL/6J mice of 6–8 weeks old and briefly sterilized by 70% ethanol. The bone marrow cells were resuspended in DMEM medium with 10% FBS, 1% P/S, and Macrophage Colony-Stimulating Factor (M-CSF) (10 ng/ml) (PeproTech, United States). The bone marrow-derived macrophages (BMDMs) were ready for further experiments after 7–10 days.

The inflammatory macrophages were established by additional 20 ng/ml IFN-γ (PeproTech) and 100 ng/ml LPS (Sigma-Aldrich, United States) treatment for 16 h. OA was prepared with DMSO (Sigma-Aldrich) as a stock solution of 50 mmol/L.

### Cell Viability

The cytotoxic effects of OA to RAW 264.7 cells were evaluated by the Methyl Thiazolyl Tetrazolium (MTT) assay, as previously described (Huang et al., 2020). In general, the seeded RAW 264.7 cells were incubated with OA and IFN-γ/LPS for 16 h, mixing with MTT, and then assayed for cell viability. The absorbance was monitored by a microplate reader at the wavelength of 490 nm (Supplementary Figure 1).

### Histological and Immunofluorescence Staining

Epidydimal white adipose tissue (eWAT) and inguinal white adipose tissue (iWAT) were fixed in 4% paraformaldehyde, embedded in paraffin after dehydration with a series of ethanol solution, and cut into slides with the thickness of 5 μm, then stained with hematoxylin and eosin (H&E). AdipoCount 1.1 was used to calculate the adipocyte area.

Immunofluorescence was performed to evaluate the macrophages recruitment to adipose tissues by immune-staining. The sections were heated in citric acid repair solution for antigen repair and then blocked with 5% normal donkey serum for 2 h. Shook off the serum and added F4/80 antibody (PBST dilution: 0.1% Tween-20 and 0.5% BSA, 1:100 dilution) dropwise, and incubated the sections in a wet box at 4°C overnight (>8 h). The next day, took out the wet box and rewarming for more than 30 min, rinsed the sections in PBS, added the corresponding fluorescent secondary antibody (1:500 dilution) and incubate it in a wet box at room temperature for 1 h. Rinsed the sections in PBS again and stained the nuclei with DAPI (4′,6-Diamidino-2-28 phenylindole dihydrochloride) for 10 min at room temperature. Fully rinsed the sections in PBS, dried the remaining PBS buffer solution, covered with 50% glycerol (diluted with PBS), and applied nail polish around the cover slides to block the air. Immediately observed the sections under a fluorescence microscope (Olympus, Japan).

### Enzyme-Linked Immunosorbent Assay and Biochemical Determination

Serum insulin levels were determined by ELISA kit (Millipore, United States) and the standard operation steps were according to the manufacturer’s protocol. Serum TG, FFAs, and T-CHO levels (Nanjing Jiancheng Company, China), and SOD, Gpx activities (Beyotime Biotechnology, China) were measured using commercial kits according to the manufacturer’s instructions. Optical density (OD) was determined on a microplate reader. HOMA-IR index = [fasted insulin (μIU/ml) × fasted glucose (mmol/L)]/22.5 (Neuschwander-Tetri, 2010). Adipo-IR index = fasted insulin (mmol/L) × fasted NEFA (pmol/L) (Musso et al., 2012).

### Quantitative Real-Time PCR

Total RNA was isolated from eWAT or cells using TRIzol Reagent (Thermo Scientific, 15596026, United States). For qRT-PCR, 1 μg total RNA from each sample was reverse-transcribed by using a Revert Aid first-strand cDNA synthesis kit (Thermo Scientific, 00698284, United States). The cDNA products were amplified using Quantstudio3/5 (Thermo Scientific, United States) real-time PCR instrument with the Power SYBR Green PCR Master Mix (Thermo Scientific, 00736756, United States). The expression levels of target genes were calculated using the 2^−ΔΔCT^ method with normalization to the standard housekeeping gene 18s, and expressed as relative mRNA levels compared with internal control. Primers used for qRT-PCR are shown in Table 1.

**TABLE 1:** Primer sequences (5′ to 3′).

Gene	Forward	Reverse
18s	CGC​CGC​TAG​AGG​TGA​AAT​TCT	CAT​TCT​TGG​CAA​ATG​CTT​TCG
F4/80	CTT​TGG​CTA​TGG​GCT​TCC​AGT​C	GCA​AGG​AGG​ACA​GAG​TTT​ATC​GTG
iNOS	CAG​AGG​ACC​CAG​AGA​CAA​GC	TGC​TGA​AAC​ATT​TCC​TGT​GC
MCP1	CTG​GAT​CGG​AAC​CAA​ATG​AG	CGG​GTC​AAC​TTC​ACA​TTC​AA
IL-6	GAC​AAC​CAC​GGC​CTT​CCC​TAC	TCA​TTT​CCA​CGA​TTT​CCC​AGA
TNFα	CGT​CGT​AGC​AAA​CCA​CCA​A	GGG​CAG​CCT​TGT​CCC​TTG​A
MRC1	CTC​TGT​TCA​GCT​ATT​GGA​CGC	TGG​CAC​TCC​CAA​ACA​TAA​TTT​GA
IL-10	GGA​CAA​CAT​ACT​GCT​AAC​CG	TTC​ATG​GCC​TTG​TAG​ACA​CC
Caspase-1	CCT​TCA​TCC​TCA​GAA​ACA​AAG​G	CAT​TAT​TGG​ATA​AAT​CTC​TGA​AGG
IL-1β	GCT​GCT​TCC​AAA​CCT​TTG​ACC	GAG​TGA​TAC​TGC​CTG​CCT​GAA
IL-18	GAC​TCT​TGC​GTC​AAC​TTC​AAG​G	CAG​GCT​GTC​TTT​TGT​CAA​CGA

### Western Blot

Cells were lyzed in cell lysis buffer on ice for 30 min. The tissue samples were sonicated (70 Hz, 90 s) in cell lysis buffer, followed by an additional incubation on ice for 20 min. Then the lysates were centrifuged at 4°C for 15 min at the speed of 12,000 rpm. The protein in the collected supernatant was degenerated under 100°C and then quantitated. Equal amounts of protein samples were loaded on SDS-PAGE gels, separated by electrophoresis, and transferred onto PVDF membranes. After being blocked with 5% skim milk, the membranes were incubated over-night at 4°C with the primary antibodies and 1 h at room temperature with appropriate secondary HRP-conjugated antibodies. Antibodies are shown in Table 2.

**TABLE 2:** Antibodies for WB.

Antibody	Source	Company	Catalog no.
β-actin	Rabbit	Cell Signaling Technology	#4967
p-Akt	Rabbit	Cell Signaling Technology	#4058
Akt	Rabbit	Cell Signaling Technology	#9272
p-HSL	Rabbit	Novus	NBP3-05459
HSL	Rabbit	Cell Signaling Technology	#4107
p-JNK	Rabbit	Cell Signaling Technology	#4668
JNK	Rabbit	Cell Signaling Technology	#9252
p-ERK	Rabbit	Cell Signaling Technology	#9101
ERK	Rabbit	Cell Signaling Technology	#9102
p-p38 MAPK	Rabbit	Cell Signaling Technology	#9211
P38 MAPK	Rabbit	Cell Signaling Technology	#9212
Caspase-1 (tissue)	Mouse	Santa Cruz	sc-56036
Caspase-1 (cell)	Rabbit	Proteintech	22915-1-AP
VDAC	Rabbit	Cell Signaling Technology	#4661
IL-1β	Hamster	Santa Cruz	sc-12742

### Flow Cytometry Analysis

Adipose tissues were minced in PBS containing 0.075% collagenase (Sigma-Aldrich, C2139, United States). After incubated at 37°C for 30 min and filtrated with 100 mesh filter, cell suspensions were centrifuged at 1,500 rpm for 5 min to remove adipocyte. Isolated stromal vascular fraction (SVF) pellet was collected from the bottom. The SVF pellet was resuspended in PBS containing 3% BSA, then red blood cell lysis buffer was added and incubated for 3 min. After washing in 3% BSA, bottom cells were incubated with Fc-Block (CD16/32, 12-0161-85, ebioscience) for 20 min at 4°C. Antibodies against CD45-FITC (11-0451-82, ebioscience), F4/80-PE (123110, Biolegend), CD11b-PerCP/Cy5.5 (101227, Biolegend), CD206-APC (141707, Biolegend) and CD11c-APC (117310, Biolegend) were added, and incubated for 20 min followed by washing in PBS containing 3% BSA. Data analysis and compensation were performed using BD Accuri C6 Plus.

RAW264.7 macrophages were digested with trypsin and terminated with PBS containing 3% BSA, then centrifuged, resuscitated and incubated with Fc-Block. The following steps were the same as above.

### *In Vitro* Chemotaxis Assay

For the migration assay, 100,000 BMDMs were placed in the upper chamber of an 8 μm polycarbonate filter (24-transwell format; Corning, United States), whereas the corresponding conditioned medium was placed in the lower chamber. After 16 h, cells were fixed in formalin, stained with crystal violet and observed under the microscope.

### Mitochondrial Reactive Oxygen Species Determination

Mitochondrial ROS level was determined using MitoSOX™ Red mitochondrial superoxide indicator (Invitrogen, United States) and FCM analysis. The specific operation steps were according to the manufacturer’s protocol.

### Statistical Analysis

All data are presented as means ± SEM. Mean value differences between two groups were assessed by two-tailed Student’s t-test. *p* values less than 0.05 were considered to be statistically significant. Statistical analyses were performed with Graph Pad Prism 8.

## Results

### Oleanolic Acid Significantly Improves Metabolic Dysfunction in Obese Mice Induced by High-Fat Diet

The obese murine model was applied here to study the effect of OA on obesity-related metabolic dysfunction. C57BL/6J mice were fed with HFD for 12 weeks, and the mice body weight increased significantly, the impaired glucose tolerance suggested establishment of the obesity model, compared with the mice fed with ND (Supplementary Figure 2). Then the obese mice were intragastric administrated with vehicle, 25 mg/kg or 50 mg/kg OA for 4 weeks. The tested OA concentration was adapted from previous studies that used doses of 20, 40, 250 mg/kg/day, or 50 mg/kg/3 days in mice (Wang et al., 2015; Su et al., 2018; Nakajima et al., 2019), and 5–100 mg/kg/day in rats (Ying et al., 2014; Lee et al., 2016; Matumba et al., 2019).

**FIGURE 1:**
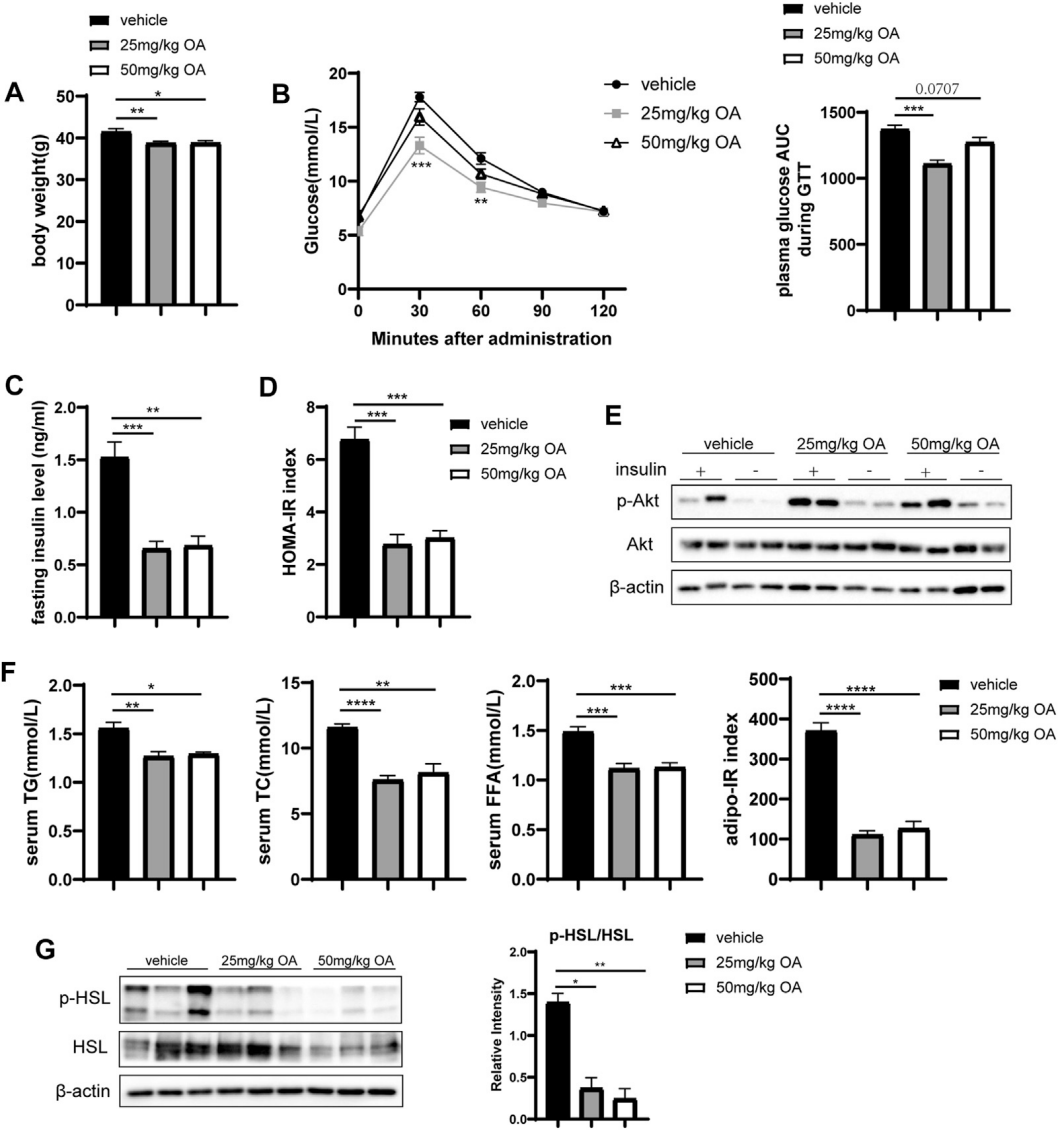
OA improves glucose and lipid metabolism and alleviates diet-induced IR. **(A)** Body weight of mice in vehicle, 25 mg/kg OA, 50 mg/kg OA group at the end of the experiment (*n* = 7). **(B)** Glucose tolerance test (GTT) in mice after 4 weeks of OA treatment (*n* = 7). **(C)** Fasting plasma insulin levels (*n* = 7). **(D)** HOMA-IR index (*n* = 7). **(E)** Western blots of phospho-Ser473 Akt (p-Akt), and Akt in eWAT of mice. **(F)** Plasma concentrations of triglyceride, total cholesterol, FFA at baseline (fasted), and Adipo-IR index (*n* = 5–7). **(G)** Western blots of phospho-Ser660 HSL (p-HSL), and HSL in the eWAT of mice. *p < 0.05, **p < 0.01, ***p < 0.001, ****p < 0.0001.

OA administration in obese mice lowered the bodyweight (Figure 1A) while improved glucose tolerance (Figure 1B), fasting insulin level (Figure 1C) and HOMA-IR index (Figure 1D). Consistently, OA administration also induced activation of the AKT pathway, which is considered as a marker event of insulin sensitivity improvement (Figure 1E). Moreover, OA decreased basal plasma concentrations of total cholesterol (TC), triglyceride (TG) and FFA (Figure 1F). In addition, the Adipo-IR index (Figure 1F) was decreased by OA treatment. Obesity-related IR can up-regulate lipolysis (Degerman et al., 2003) and increase FFA levels. Figure 1G showed the ratio of phosphorylated hormone sensitive lipase (p-HSL) to HSL, which usually used as the indicator of adipose lipolysis, was down-regulated by OA treatment. The 25 mg/kg dose of OA treatment had maximal effect on these improvements. These data showed that OA could improve glucose and lipid metabolism in HFD-induced obese mice. Notably, the improvement in inflammation, glucose tolerance, and insulin sensitivity in the OA-treated mice also sustained in the body-weight matched groups (Supplementary Figure 3).

### Oleanolic Acid Treatment Reduces the Adipocyte Hypertrophy and the Macrophages Infiltration Into Epidydimal White Adipose Tissue

**FIGURE 2:**
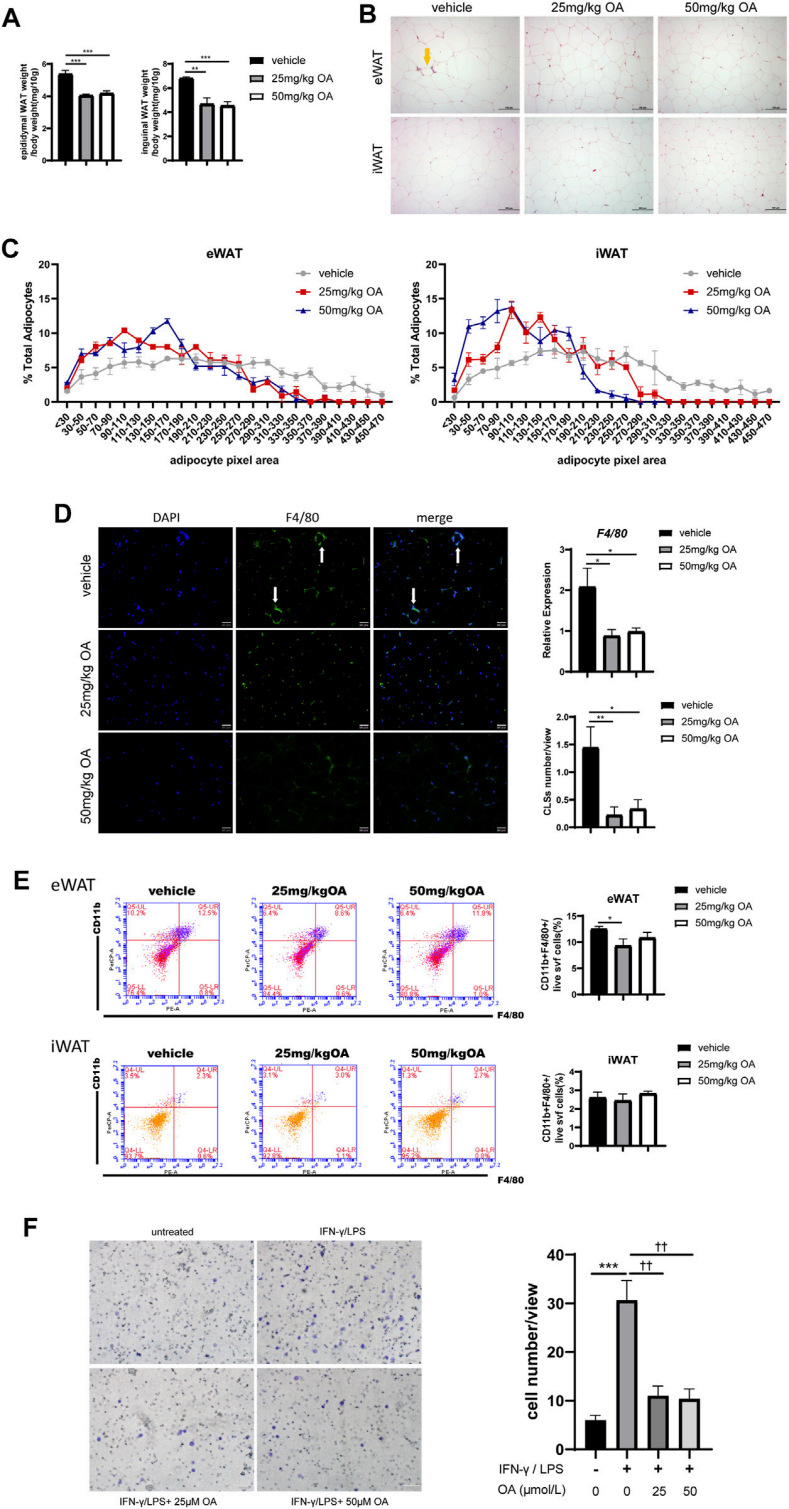
OA diminishes adipocyte size and reduces macrophage infiltration to eWAT in DIO mice. **(A)** Ratio of eWAT/iWAT to body weight of mice (*n* = 7). **(B)** HE staining results of eWAT and iWAT. Scale bars = 100 µm. **(C)** Statistics of the adipocyte area (*n* = 51–76). **(D)** F4/80 immunofluorescence staining, mRNA expression (*n* = 5) and CLSs statistics (*n* = 9) of eWAT. Scale bars = 50 µm. **(E)** Representative plots and statistics of FCM analysis: total ATMs to the SVF of eWAT and iWAT (*n* = 5). **(F)** Transwell results of BMDMs, scale bar = 100 µm (*n* = 6).*p < 0.05, **p < 0.01, ***p < 0.001, ****p < 0.0001 vs. vehicle or control incubations, ^††^ *p* < 0.01 vs. IFN-γ/LPS stimulated incubations.

Figure 1 has shown that OA treatment improves IR, thus we next tried to underline how OA treatment achieves its effects on IR. It is known that changes in adipocyte morphology and infiltration of macrophages in adipose tissue contribute to IR development (Xu et al., 2020), we then examined the effect of OA on adipocyte morphology and macrophage infiltration. As shown in Figure 2A, OA treatment reduced the ratio of adipose tissue weight to body weight, and significantly decreased the adipocyte size in HFD-treated mice (Figures 2B,C). HE staining and immunofluorescence showed fewer CLSs in the eWAT after OA treatment, which suggested a decreased macrophage accumulation (Figures 2B,D). The mRNA expression of F4/80 (a macrophage marker) in eWAT was evaluated by qPCR, and the proportion of F4/80^+^ cells in eWAT was measured by FCM. The results showed a decreasing trend of macrophage infiltration by OA treatment (Figures 2D,E).

Macrophages infiltrating adipose tissue are generally chemotactic from peripheral blood. In order to further confirm whether OA could reduce the chemotaxis of macrophages, the effect of OA on macrophage migration under inflammatory condition was assayed using trans-well chemotaxis assay *in vitro*. As shown in Figure 2F, OA significantly inhibited IFN-γ/LPS-induced macrophage migration. These data suggested that OA decrease macrophage infiltration by inhibiting chemotaxis of macrophages.

### Oleanolic Acid Attenuates Inflammation and Changes the Proportion of M1 and M2 Macrophages in Adipose Tissue of High-Fat Diet Mice

**FIGURE 3:**
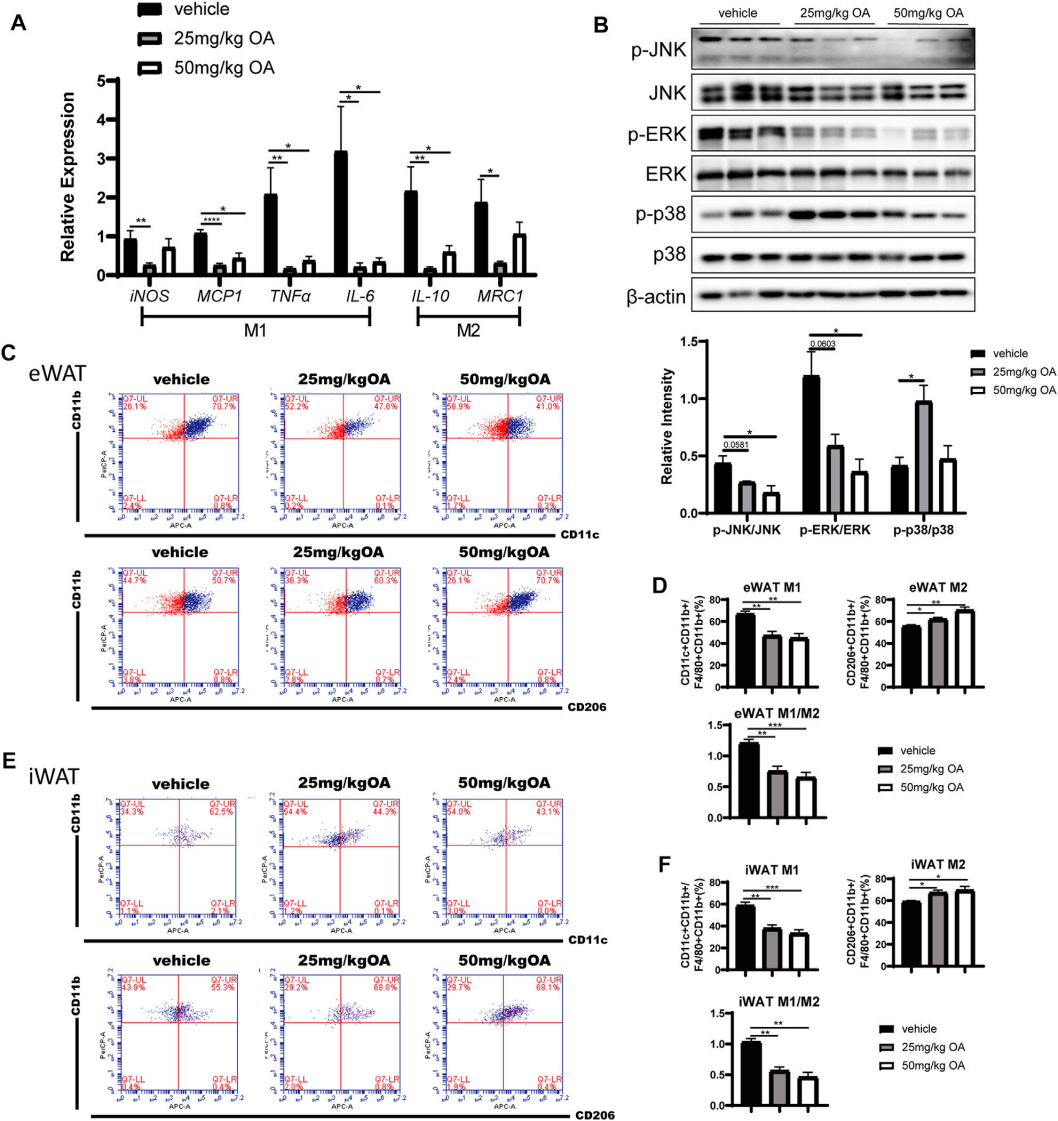
OA decreases eWAT inflammation level and ratio of M1/M2 ATMs in DIO mice. **(A)** ATMs associated markers in eWAT (*n* = 5). **(B)** WB of phosphorylated p38 MAPK (p-p38 MAPK), phosphorylated JNK (p-JNK), phosphorylated ERK (p-ERK), and their total proteins in eWAT of mice. **(C–F)** FCM analysis of the SVF of eWAT and iWAT of mice (*n* = 5). **(C, E)** Representative plots of FCM analysis: proportion of M1 or M2 macrophages to ATMs. **(D, F)** Statistics of FCM analysis. Data are percentages of M1 ATMs, percentages of M2 ATMs, and M1/M2 ratios. *p < 0.05, **p < 0.01, ***p < 0.001, ****p < 0.0001.

An significant increase in ATM infiltration is often observed in eWAT rather than iWAT in the process of obesity (Gómez-Ambrosi et al., 2004; Cancello et al., 2005; Amano et al., 2014). Consequently, infiltrated ATM induced expression of inflammatory markers that play key regulatory roles in the development of obesity-related IR (Dong et al., 2014). Here we next investigated whether OA treatment can impact inflammation in eWAT. The qPCR results showed that pro-inflammatory markers derived from M1 macrophages (iNOS, MCP1, TNFα) were significantly down-regulated in eWAT of OA administrated mice compared with the vehicle treated mice (Figure 3A). The decreased expression of M2 macrophage related genes in eWAT (Figure 3A) may be due to the decrease in the total number of infiltrated macrophages in adipose tissue after OA treatment. These findings were associated with attenuated phosphorylation of c-Jun N-terminal kinase (JNK) and extracellular signal-regulated kinase (ERK) (the key proteins in MAPK signaling pathway) in eWAT of diet-induced obesity (DIO) mice (Figure 3B).

To explain how inflammation is down-regulated in eWAT of OA-administrated mice, we tested whether this decrease is due to regulation of macrophages polarization in eWAT. FCM analysis showed that the proportion of M1 ATMs decreased in WAT of obese mice with OA treatment, whereas the proportion of M2 ATMs increased, the ratio of M1/M2 decreased significantly (Figures 3C–F). The polarity transition of ATMs could lead toward an anti-inflammatory phenotype.

Taken together, these results suggested that OA treated mice showed attenuated inflammation and decreased M1/M2 ratio in WAT of DIO mice.

### Oleanolic Acid Decreases Inflammation and Inhibits M1 Polarization Induced by IFN-γ/LPS in Macrophages *In Vitro*

**FIGURE 4:**
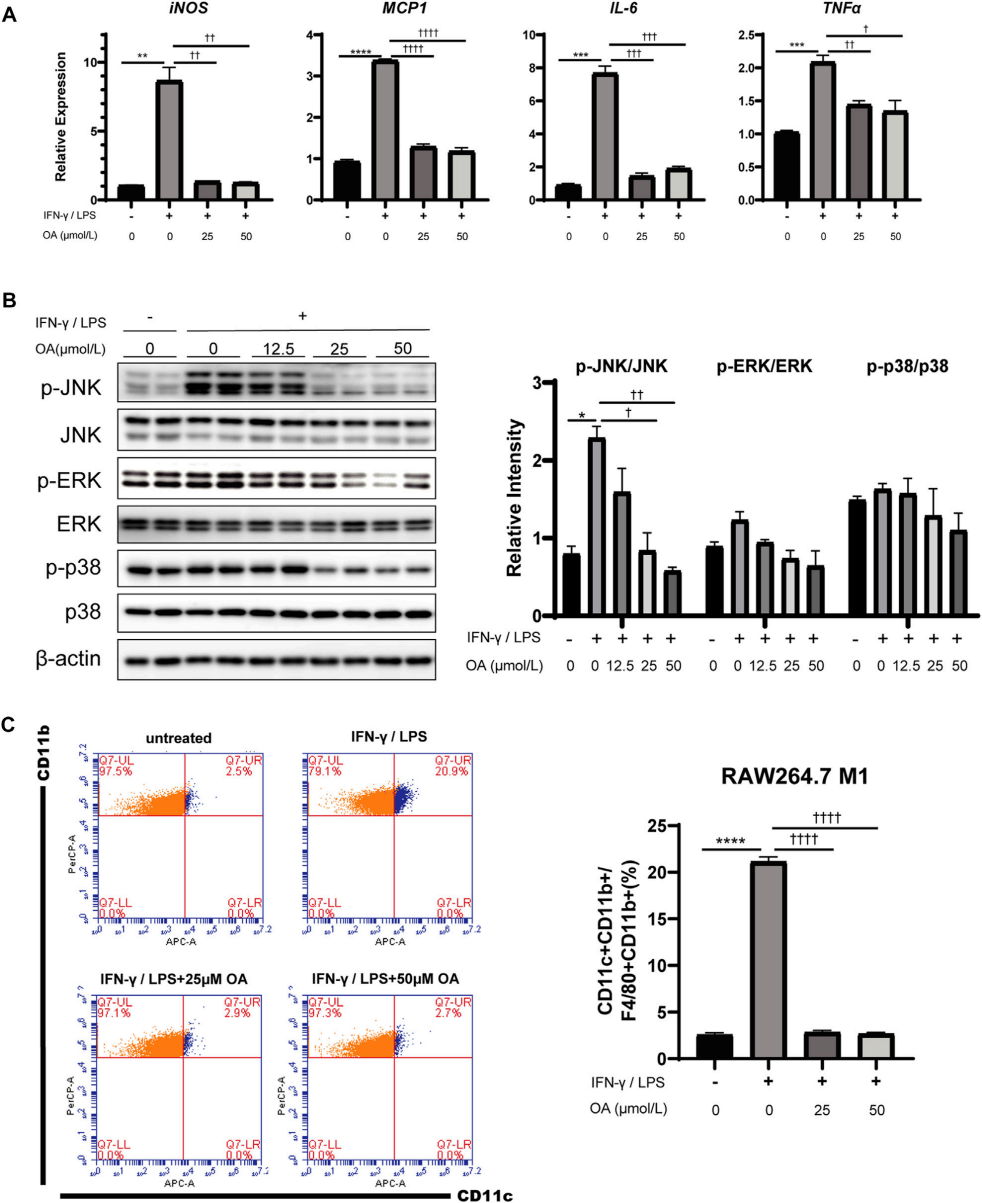
OA reduces inflammation and M1 polarization in RAW264.7 macrophages stimulated by IFN-γ/LPS. **(A)** OA suppresses the increase of IFN-γ/LPS induced M1 markers mRNA expression in RAW264.7 macrophages (*n* = 3). **(B)** WB of phosphorylated p38 MAPK (p-p38 MAPK), phosphorylated JNK (p-JNK), phosphorylated ERK (p-ERK), and their total proteins in RAW264.7 macrophages. **(C)** FCM analysis of the M1 ratio of RAW264.7 macrophages (n = 3). **p < 0.01, ***p < 0.001, ****p < 0.0001 vs. control incubations, ^†^ *p* < 0.05, ^††^ *p* < 0.01, ^†††^ *p* < 0.001, ^††††^ *p* < 0.0001 vs. IFN-γ/LPS stimulated incubations.

Given the association between OA treatment and ATM polarization in obese mice, RAW264.7 macrophages and bone marrow-derived macrophages (BMDMs) were engaged to determine whether OA directly regulates macrophage activation and/or polarization. Consistently with results *in vivo*, the expression levels of M1 marker genes (iNOS, MCP1, IL-6 and TNFα) were decreased in these macrophages stimulated with the combination of IFN-γ/LPS and OA (Figure 4A; Supplementary Figure 4B), and the phosphorylation of JNK, ERK and p38 were also inhibited by the treatment as expected (Figure 4B; Supplementary Figure 4A). Furthermore, flow cytometry analysis showed that OA significantly inhibited the IFN-γ/LPS induced M1 polarization (Figure 4C).

### Oleanolic Acid Resists the Activation of NLRP3 Inflammasome by Blocking Voltage Dependent Anion Channels and Reducing Reactive Oxygen Species Production

To investigate how OA reduce adipose tissue inflammation and inhibit macrophage M1 polarization, we further tested whether it depends on regulating activation of the NLRP3 inflammasome. The NLRP3 inflammasome (NLRP3/ASC/caspase-1 complex) is a key player of inflammation and M1 macrophage polarization (Ślusarczyk et al., 2018), and plays a central role in the induction of obesity and IR (Stienstra et al., 2011; Vandanmagsar et al., 2011).

**FIGURE 5:**
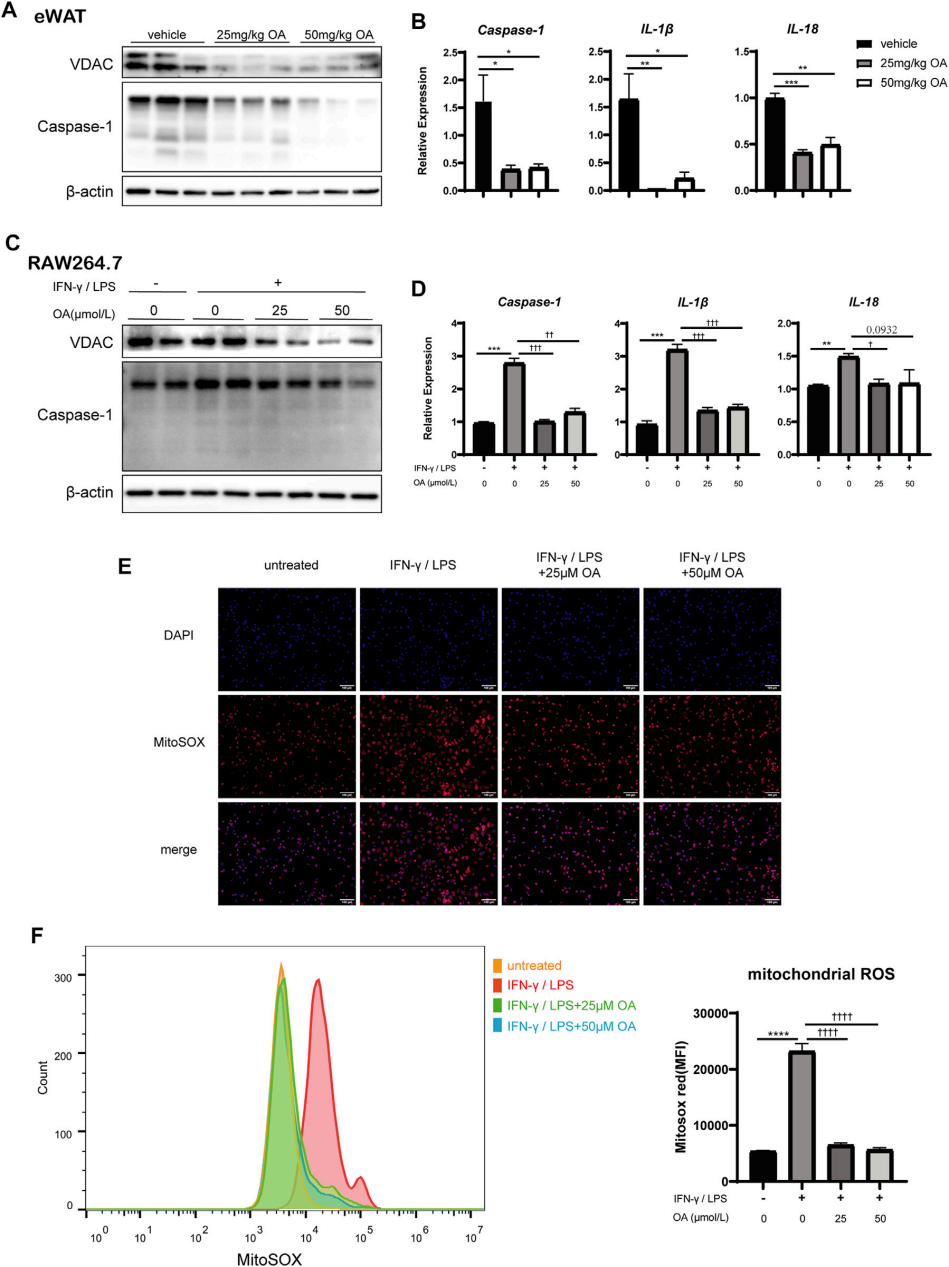
OA decreases the expression of VDAC, the activation of NLRP3 inflammasome and the production of mitochondrial ROS *in vitro* and/or *in vivo*. **(A)** WB of VDAC, Caspase-1, IL-1β in eWAT of mice. **(B)** qPCR results of Caspase-1, IL-1β and IL-18 in eWAT (*n* = 5). **(C)** WB of VDAC, Caspase-1 in RAW264.7 macrophages. **(D)** qPCR results of Caspase-1, IL-1β and IL-18 in RAW264.7 macrophages (*n* = 3). **(E)** Fluorescence staining of mitochondrial ROS in RAW264.7 macrophages. **(F)** Fluorescence intensity of MitoSOX analyzed by FCM in RAW264.7 macrophages (*n* = 3–4). *p < 0.05, **p < 0.01, ***p < 0.001, ****p < 0.0001 vs. vehicle or control incubations, ^†^ *p* < 0.05, ^††^ *p* < 0.01, ^†††^ *p* < 0.001, ^††††^ *p* < 0.0001 vs. IFN-γ/LPS stimulated incubations.

We firstly examined the expression of caspase-1, which was the effector of NLRP3 inflammasome, in RAW264.7, BMDMs and mice eWAT. The data showed that the up-regulation of caspase-1 induced by IFN-γ/LPS or DIO was inhibited significantly by OA treatment (Figures 5A–D; Supplementary Figures 5A,B). Consistently, the levels of IL-1β and IL-18, the inflammatory cytokines processed by inflammasome, were also decreased significantly with OA treatment (Figures 5B,D; Supplementary Figure 5B). Our results suggested that OA could resist the activation of NLRP3 inflammasome.

Due to ROS production in macrophages triggers the activation of NLRP3 inflammasome (Dostert et al., 2008; Eisenbarth et al., 2008), the levels of the mitochondrial ROS was detected. Figures 5E,F revealed that OA attenuated the up-regulation of ROS production induced by IFN-γ/LPS stimulation in RAW 264.7 cells.

Voltage dependent anion channels, the most abundant proteins of the outer mitochondrial membrane (Colombini, 2004), is known to regulate mitochondrial ROS production (Da-Silva et al., 2004) and associated with the NLRP3 inflammasome (Wolf et al., 2016). Previous investigators had noted that the knockdown of the VDAC somehow blocks NLRP3 inflammasome activation (Zhou et al., 2011). With IFN-γ/LPS stimulation *in vitro* or DIO *in vivo*, VDAC protein levels were up-regulated, which is consistent with ROS production. However, OA attenuated the increase of VDAC protein levels (Figures 5A,C; Supplementary Figure 5A).

**FIGURE 6:**
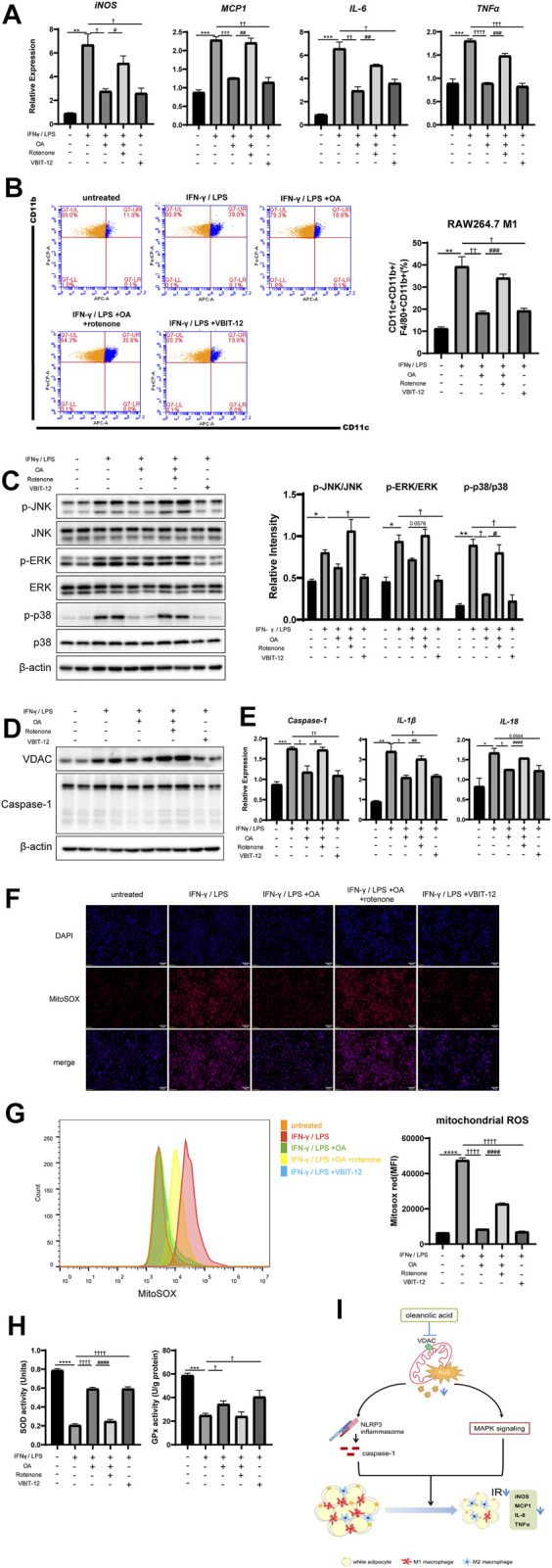
Rotenone neutralized the anti-inflammatory effect of OA, while VBIT-12 showed a similar effect with OA in RAW264.7 macrophages. **(A)** Rotenone attenuated the inhibitory effect of OA on the expression of M1 marker mRNA in RAW264.7 macrophages induced by IFN-γ/LPS, while VBIT-12 had a similar effect to OA. (*n* = 3). **(B)** FCM analysis of the M1 ratio of RAW264.7 macrophages (*n* = 3). **(C)** WB of phosphorylated p38 MAPK (p-p38 MAPK), phosphorylated JNK (p-JNK), phosphorylated ERK (p-ERK), and their total proteins in RAW264.7 macrophages. **(D)** WB of VDAC, Caspase-1 in RAW264.7 macrophages. **(E)** qPCR results of Caspase-1, IL-1β and IL-18 in RAW264.7 macrophages (*n* = 3). **(F)** Fluorescence staining of mitochondrial ROS in RAW264.7 macrophages. **(G)** Fluorescence intensity of MitoSOX analyzed by FCM in RAW264.7 macrophages (*n* = 5). **(H)** The activities of SOD and GPx. (*n* = 3). **(I)** A summary of our current findings. Oleanolic acid alleviates inflammation and regulates macrophage polarization in adipose tissue of obese mice to improve insulin resistance, at least in part by inhibiting VDAC and thus reducing mitochondrial ROS, thereby negatively regulating the activation of NLRP3 inflammasome and MAPK signaling pathway. *p < 0.05, **p < 0.01, ***p < 0.001, ****p < 0.0001 vs. control incubations, ^†^ *p* < 0.05, ^††^ *p* < 0.01, ^†††^ *p* < 0.001, ^††††^ *p* < 0.0001 vs. IFN-γ/LPS stimulated incubations, ^#^ *p* < 0.05, ^##^ *p* < 0.01, ^###^ *p* < 0.001, ^####^ *p* < 0.0001 vs. the combination of IFN-γ/LPS and OA incubations.

To further determine whether OA improves inflammation and regulates macrophage polarization through VDAC and ROS, rotenone (selleck, United States, 10 μmol/L), a mitochondrial complex Ι inhibitor that can lead to the up-regulation of VDAC and robust ROS production (Zhou et al., 2011; Jiang et al., 2017), was added 6 h before cell harvest to counteract the effects of OA on VDAC expression and ROS production in RAW264.7 macrophages. We observed that the addition of rotenone up-regulated VDAC, increased the production of ROS, and significantly weakened the anti-inflammatory and the inhibition effect of the MAPK pathway and inflammasome activation of OA. In addition, inhibition of VDAC by the inhibitor VBIT-12 (selleck, United States, 20 μmol/L) in RAW264.7 macrophages treated with IFN-γ/LPS mimicked the OA’s effects (Figure 6). Endogenous antioxidant enzymes such as superoxide dismutase (SOD) and glutathione peroxidase (GPx) have been known as the main regulator in the mitochondrial ROS production, and their activation can lead to the remission of inflammatory response (Gasparrini et al., 2017; Zhao et al., 2020). We found that OA increased the activities of SOD and GPx in RAW264.7 macrophages stimulated by IFN-γ/LPS, while rotenone attenuated this effect. As expected, VBIT-12 also increased the activities of SOD and GPx (Figure 6H). Taken together, these data supported that OA could inhibit the activation of NLRP3 inflammasome by reducing ROS production and VDAC expression.

## Discussion

The association of obesity, adipose tissue inflammation, and metabolic diseases makes the inflammatory pathways an appealing direction for designing the novel treatment of obesity-related metabolic complications (Belkina and Denis, 2010; Gregor and Hotamisligil, 2011; Zatterale et al., 2019). Consistent with previous reports (Lee et al., 2013; Ying et al., 2014; Zohra et al., 2018), our research showed that OA can improve IR (Figure 1) and has anti-inflammatory effects (Figures 3A,B, 4B,C; Supplementary Figure 4). For the first time, we reported that OA can improve adipose tissue inflammation by regulating macrophage infiltration and polarization in therapeutic administration mice model.

Macrophages are known to play an important role in obesity-related metabolic diseases progression and contribute to the development of diabetic complications in concert with endothelial cells and adipocytes (Giacco and Brownlee, 2010; Pitocco et al., 2013). ROS is essential for the induction and maintenance of polarization of M1 type macrophages, and it activates multiple pro-inflammatory pathways including MAPK and NLRP3 inflammasome (Kohchi et al., 2009; Sorbara and Girardin, 2011; Gan et al., 2016; Zhao et al., 2019). Recent studies about oxidative stress in diabetic complications assessed ROS in certain cell types such as endothelial and epithelial cells (Xiao et al., 2014; Nishikawa et al., 2015; Qi et al., 2017). However, the role of macrophage-generated ROS in obesity complications is still underappreciated. Here, our study shows that OA, a potent antioxidant, can suppress M1-macrophage polarization by eliminating ROS that act as a second messenger regulating the IFN-γ/LPS-stimulated MAPK pathway and activation of NLRP3 inflammasome (Figures 4–6).

Previous studies show that MAPK is an important signaling pathway that regulates inflammation and is associated with metabolic dysfunctions in obesity and diabetes (Gehart et al., 2010). For instance, ERK modulates inflammatory activation of macrophages to inhibit beige adipogenesis in obesity (Chung et al., 2017), p38 regulates inflammation and insulin signaling (Nandipati et al., 2017), JNK promotes obesity-induced IR by regulating ATMs M1 polarization in WAT (Han et al., 2013). In this study, p-JNK and p-ERK was down-regulated but p-p38 was up-regulated in eWAT of mice treated with OA compared with the one of the control mice treated with vehicle (Figure 3B), while *in vitro*, the expression levels of all three phosphorylated kinases in IFN-γ/LPS stimulated macrophages were down-regulated by OA treatment (Figure 4B; Supplementary Figure 4A). This difference between *in vivo* and *in vitro* might be due to the fact that there are more cell types involved at the tissue level. As in adipose tissue, there are adipocytes and many other cell types in addition to macrophages, while only macrophages are used in *in vitro* experiments. The OA-dependent regulation of p38 phosphorylation in non-macrophage cells may be different. Another reason could be that the stimulating factors triggering inflammation *in vivo* and *in vitro* are different. Besides, although p-p38, p-ERK and p-JNK all play important roles in inflammation, p-JNK which is more important for macrophage polarization (Vallerie et al., 2008; Han et al., 2013), was down-regulated both *in vivo* and *in vitro*. In addition to the MAPK signaling pathway and NLRP3 inflammasome, it is not excluded that OA acts through other downstream pathways to regulate obesity-induced inflammation.

The main source of a diverse variety of ROS in most cells is mitochondria (Figueira et al., 2013; Ganeshan and Chawla, 2014), and the mitochondria membrane proteins take important roles in ROS production. VDAC, the most abundant protein in mitochondria membrane, is reported to be ultimately required for ROS production (Messina et al., 2011). Our work revealed that OA can down-regulate VDAC and impair ROS production. Interestingly, it has been reported that VDAC inhibitors can restore insulin secretion in type 2 diabetes islet donors and prevent hyperglycemia in diabetic mice (Zhang et al., 2019). This is the first report that OA may play its role by affecting mitochondrial function. But how VDAC is regulated by OA needs to be further studied.

## Data Availability

The raw data supporting the conclusion of this article will be made available by the authors, without undue reservation.
