# Supplementary material for: Oleanolic Acid Improves Obesity-Related Inflammation and Insulin Resistance by Regulating Macrophages Activation
Source: Front Pharmacol. 2021 Jul 30;12:697483. doi: 10.3389/fphar.2021.697483 (PMC8361479; doi:10.3389/fphar.2021.697483)
Supplement: Supplementary file 1 [file DataSheet1.docx]

Supplementary Material


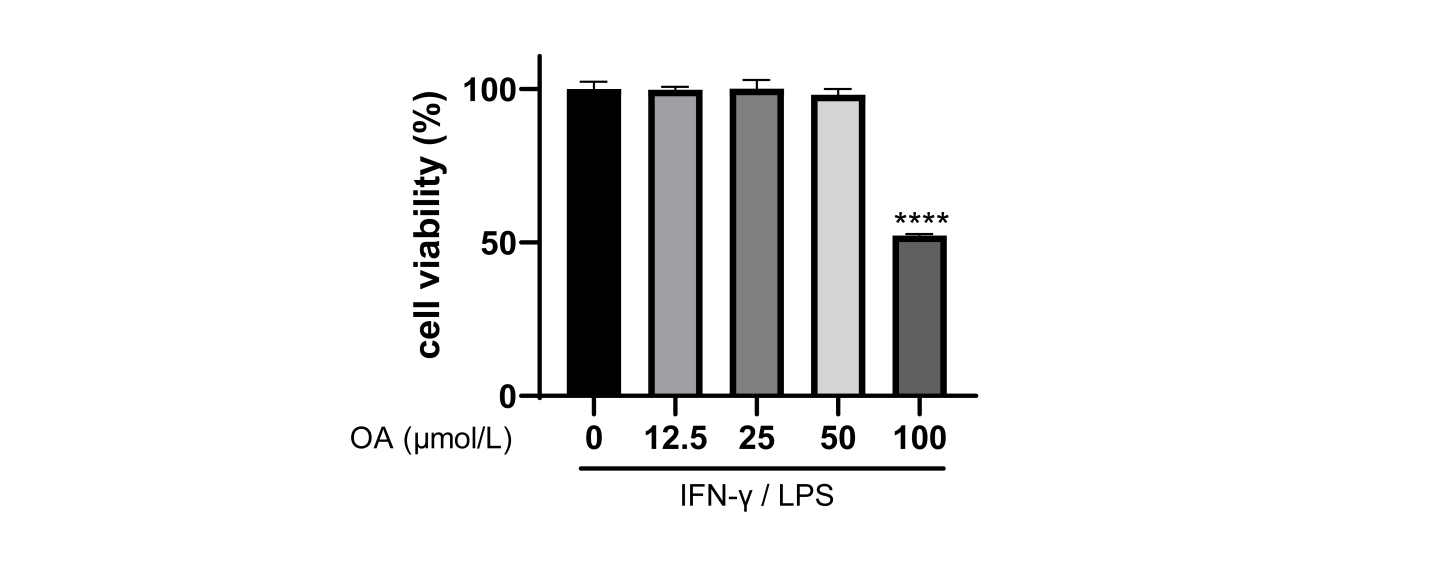


**Supplementary Figure 1.** RAW 264.7 macrophages were treated with OA (0-100 μmol/L) and IFN-γ/LPS for 24 h. Cell viability was measured by MTT assay (n = 4). ****P < 0.0001.


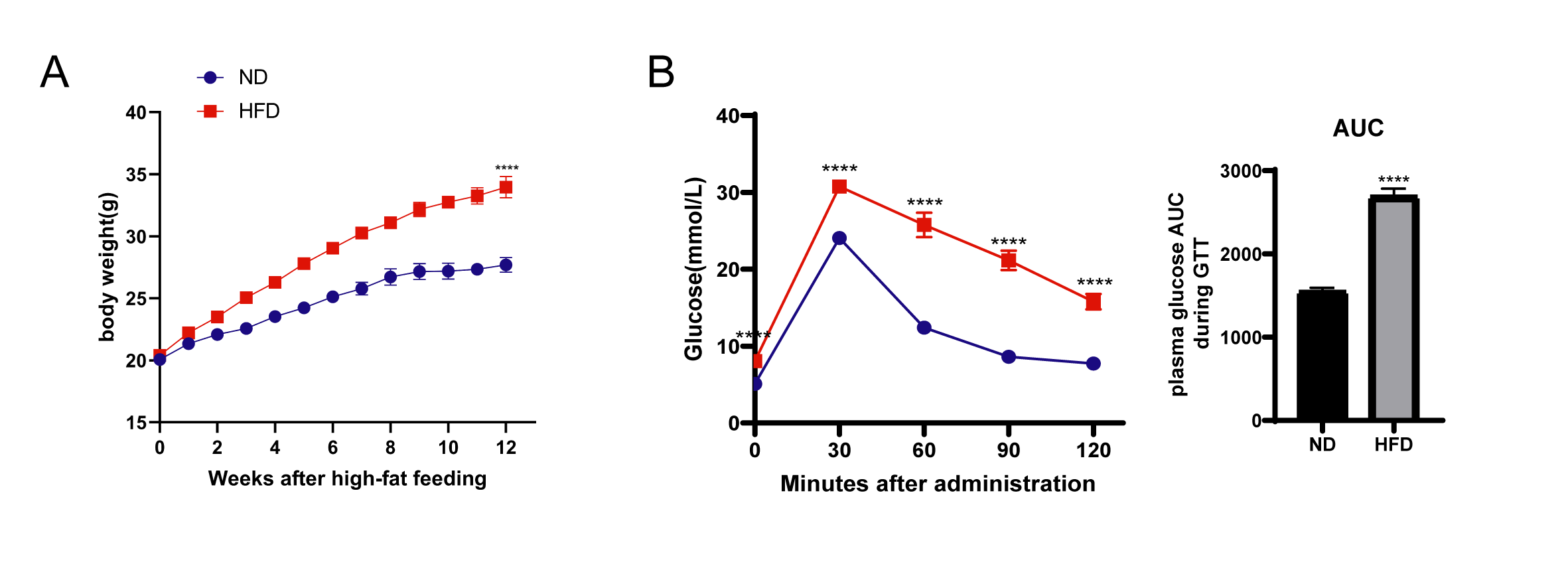


**Supplementary Figure 2.** The obesity model was successfully established in mice fed with HFD for 12 weeks. **(A)** Body weight curves of mice initiated on HFD and those on ND for the same week of age. **(B)** GTT in mice fed a HFD or ND for 12 weeks. n = 7. ****P < 0.0001.


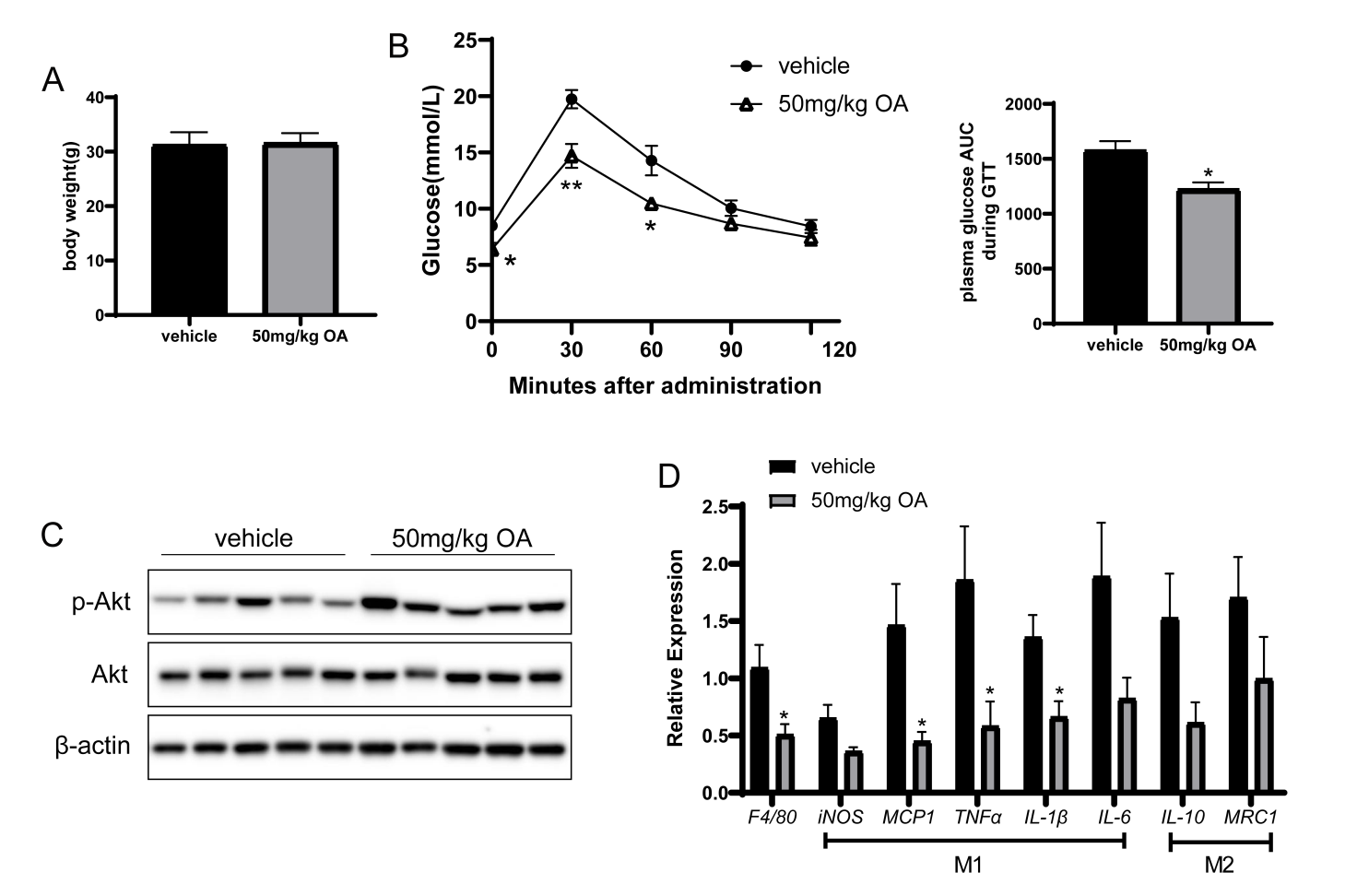


**Supplementary Figure 3.** OA improves glucose tolerance, and eWAT insulin sensitivity and inflammation in the HFD body-weight matched groups. **(A)** There was no difference in the body weight of mice fed HFD for 10 weeks and given OA or vehicle gavage for 1 week. **(B)** GTT in mice after 1 weeks of OA treatment. **(C)** Western blots of phospho-Ser473 Akt (p-Akt), and Akt in eWAT of mice. **(D)** ATMs associated markers in eWAT. n = 5. *P < 0.05, **P < 0.01.


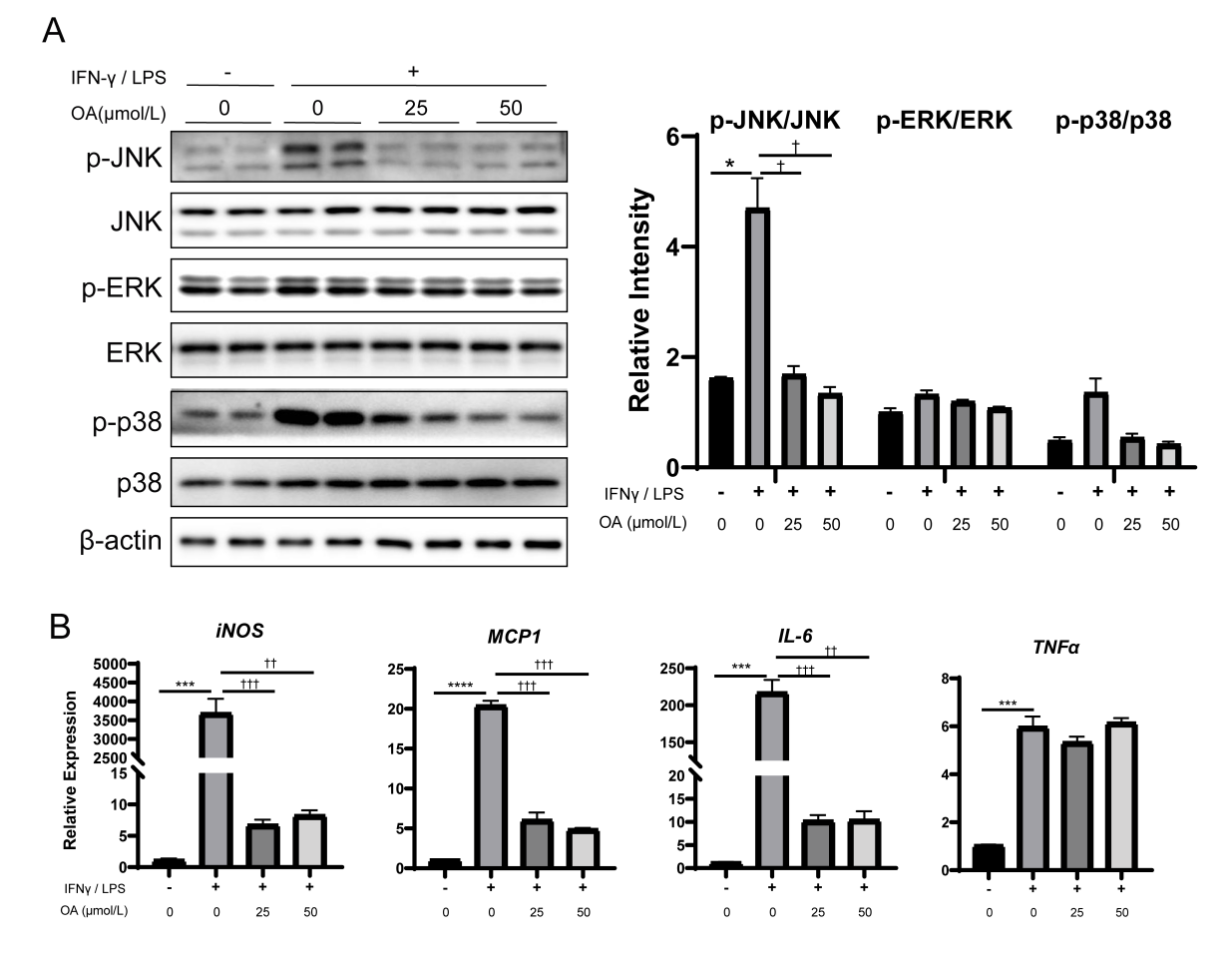


**Supplementary Figure 4.** OA inhibits inflammatory response of BMDMs induced by IFN-γ/LPS. **(A)** WB of phosphorylated p38 MAPK (p-p38), phosphorylated JNK (p-JNK), phosphorylated ERK (p-ERK), and their total proteins in BMDMs. **(B)** mRNA expression of M1 markers in BMDMs. n = 3. ***P < 0.001, ****P < 0.0001 vs. control incubations, ††P < 0.01, †††P < 0.001 vs. IFN-γ/LPS stimulated incubations.


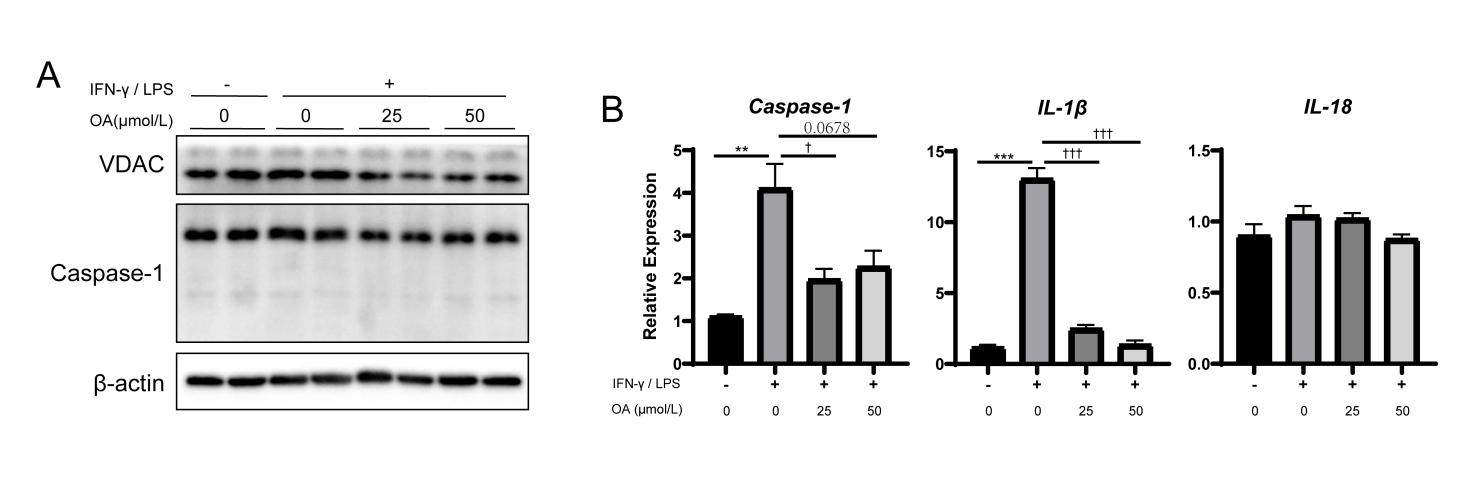


**Supplementary Figure 5.** OA inhibits IFN-γ/LPS-induced VDAC expression and NLRP3 inflammasome activation in BMDMs. **(A)** WB of VDAC, Caspase-1 in BMDMs. **(B)** qPCR results of Caspase-1, IL-1β and IL-18 in BMDMs (n = 3).**P < 0.01, ***P < 0.001 vs. control incubations, †P < 0.05, †††P < 0.001 vs. LPS stimulated incubations.
